# Racial and ethnic differences in bladder cancer diagnosis, treatment, and specialty care access among *Medicare* fee-for-service beneficiaries: a cross-sectional study

**DOI:** 10.3389/fonc.2026.1797699

**Published:** 2026-06-22

**Authors:** Atreyee Majumder, Lisa Dwyer Orr

**Affiliations:** Johnson & Johnson, Titusville, NJ, United States

**Keywords:** bladder cancer, cancer care, racial and ethnic differences, specialist access, treatment initiation

## Abstract

**Introduction:**

Bladder cancer (BC) is the second most common genitourinary malignancy, with substantial racial disparities in survival. This study assessed racial and ethnic differences across key stages of BC care among US Medicare beneficiaries.

**Methods:**

This cross-sectional study analyzed Medicare fee-for-service claims (2021–2022) and included patients with newly diagnosed BC (ICD-10-CM code C67). Differences in diagnosis, specialty care access, and treatment across racial and ethnic groups (White, Black or African American, Hispanic, Asian or Pacific Islander, American Indian or Alaska Native, Other, or Unknown) were evaluated using rate ratios (RRs), with White patients as the reference group. Median times from diagnosis to first specialist referral and to treatment initiation were also assessed. Outcomes are reported using descriptive statistics.

**Results:**

A total of 136, 172 patients met the study criteria: 116, 043 (85.2%) White, 7, 768 (5.7%) Black or African American, 5, 733 (4.2%) Hispanic, 2, 729 (2.0%) Asian or Pacific Islander, 365 (<1%) American Indian or Alaska Native, and 3, 534 (2.6%) patients of Other or Unknown race. The RRs for BC diagnosis and receipt of any treatment were below 1.0 across all racial and ethnic groups. Compared with White patients, Hispanic and Black or African American patients had less access to specialists (Hispanic: RR = 0.891; 95% confidence interval [CI], 0.865–0.918; Black or African American: RR = 0.930; 95% CI, 0.906–0.954), had lower rates of receiving any treatment (Hispanic: RR = 0.806; 95% CI, 0.765–0.850; Black or African American: RR = 0.814; 95% CI, 0.775–0.856), and experienced longer median times from diagnosis to treatment initiation (31 days for both groups vs 25 days for White patients). Hispanic and American Indian or Alaska Native patients also had longer median times from initial diagnosis to first specialist visit (26 and 28 days, respectively) compared with White patients (23 days).

**Conclusion:**

Racial disparities in BC care across the care continuum were observed. These findings can inform conversations with population health decision makers and policy-level stakeholders to identify solutions to help improve care for minoritized racial and ethnic groups. Further research is needed to understand the underlying drivers and develop evidence-based strategies to ensure equitable access to care for all patients.

## 1 Introduction

Bladder cancer is the second most common genitourinary malignancy in the United States (US), with an estimated 84, 870 new cases and 17, 420 deaths in 2025 (1). It is also one of the most expensive cancers to manage over a patient’s lifetime because of its high recurrence rate and need for ongoing surveillance (2). Bladder cancer is often diagnosed among individuals aged 65 years and older. The 5-year relative survival rate is approximately 79.0% but declines substantially with disease progression, ranging from 97.9% for *in situ* disease and 72.6% for localized disease to 40.5% for regional spread and 9.1% for distant metastasis (1).

Racial and ethnic disparities in bladder cancer incidence and outcomes are well documented (3–5). Although non-Hispanic White individuals have the highest overall incidence and mortality rates, Black patients are more often diagnosed at advanced stages and experience disproportionately worse bladder cancer-specific survival, even after accounting for disease stage (3, 6). Prior studies suggest that access- and treatment-related factors are key contributors to these disparities (7–10).

Access to specialist-directed care, in particular, is essential for effective bladder cancer management (11, 12). Delayed or lack of referral to urologists and oncologists can reduce patients’ opportunities for early detection and appropriate treatment, potentially worsening outcomes and exacerbating existing disparities (12–14). Prior studies have shown that treatment delays are associated with worse overall and cancer-specific survival (15–17). Similarly, minimizing delays in treatment initiation is critical to optimizing treatment outcomes. However, despite the importance of medical specialist involvement and minimizing delays in treatment initiation, differences in access to specialty care and in time from initial diagnosis to treatment initiation among minoritized racial and ethnic groups remain poorly characterized.

Meanwhile, recent therapeutic advances and regulatory approvals, including immunomodulatory agents, targeted therapies, and advanced drug-delivery systems, are reshaping the treatment landscape of bladder cancer (18–21). However, realizing the full benefits of these advances depends on timely diagnosis, access to specialty care, and prompt treatment initiation, which continue to be less attainable for minoritized populations. Current, population-based evidence is, therefore, needed to elucidate where disparities persist along the care continuum and to identify opportunities for intervention. In this study, we aimed to assess racial and ethnic differences across key stages of bladder cancer care, including diagnosis, medical specialist access, and time from initial diagnosis to treatment among US Medicare beneficiaries with newly diagnosed bladder cancer.

## 2 Methods

### 2.1 Study design and data source

We conducted an observational, cross-sectional study using the Medicare fee-for-service (FFS) claims data from January 1, 2021, to December 31, 2022. Racial and ethnic differences in diagnosis, access to medical specialists, and receipt of treatment interventions were evaluated using rate ratios (RRs). An RR is a measure of relative difference; a value of 1.0 indicates no difference in rates between groups, while values above or below 1.0 reflect relative differences. Times from diagnosis to specialist referral and to treatment initiation were also assessed. All analyses were performed within the Centers for Medicare & Medicaid Services (CMS) Virtual Research Data Center (VRDC), a secure, cloud-based environment that complies with CMS privacy and security requirements. Because this study used only de-identified data, institutional review board (IRB) review and oversight were not required.

### 2.2 Study population

This study included patients of any age with newly diagnosed bladder cancer, identified using the International Classification of Diseases, Tenth Revision, Clinical Modification (ICD-10-CM) diagnosis code C67. Patients were classified as having newly diagnosed bladder cancer if, during the study period, they had ≥1 inpatient, skilled nursing facility (SNF), hospice, home health, or carrier claims or ≥2 outpatient claims separated by ≥30 days, for bladder cancer; and if they had no prior history of the disease. Eligible patients were required to have continuous enrollment in Medicare Parts A and B for ≥1 year before diagnosis.The initial bladder cancer diagnosis date was defined as the index date.

For the treatment analysis, eligible patients were also required to have ≥1 relevant procedure code recorded in any setting, including inpatient, SNF, hospice, home health, or outpatient claims, within 30 days before or after the index date, and continuous Part D enrollment from December 2020 to June 2023. For the medical specialist-access analysis, eligible patients were required to have ≥1 visit with a specialist in one of the following specialty fields: urology, oncology, medical oncology, surgical oncology, or radiation oncology, identified via National Provider Identifiers and matched with the Definitive Healthcare database.

Patients were excluded if their first bladder cancer claim was outside the study period, or if their ICD-10-CM diagnosis code C67 co-existed with a rule-out code Z03.89. Race and ethnicity categories were classified as White, Black or African American, Hispanic, Asian or Pacific Islander, American Indian or Alaska Native, “Other” (i.e., beneficiaries not classified into the standard categories), or Unknown, as defined by CMS enrollment records.

### 2.3 Outcome measures

The outcomes of interest were as follows: (1) RRs for initial bladder cancer diagnosis, (2) RRs for receipt of pharmacological and/or non-pharmacological interventions, (3) RRs for specialist visits, (4) time from initial diagnosis to specialist care, and (5) time from initial diagnosis to treatment initiation. Pharmacological interventions included oral medication, chemotherapy, immunotherapy, and targeted therapy. Non-pharmacological interventions included surgery (cystectomy and transurethral resection of bladder tumor [TURBT]) and radiation therapy. Specialist care consisted of care provided by urologists and oncologists, including medical oncologists, surgical oncologists, and radiation oncologists. Time-to-specialist care and time-to-treatment initiation were defined as the number of days from the index date to the first documented specialist visit or treatment, respectively.

All outcomes were reported using descriptive statistics. Differences in diagnosis, receipt of treatment, and specialist care were reported as RRs with 95% confidence intervals (CIs), using White patients as the reference group. Times from initial diagnosis to specialist care and to treatment initiation were summarized using medians and interquartile ranges (IQRs). All analyses were performed using R (version 4.3.2) in Databricks within the CMS VRDC.

*Sensitivity analyses*

Two sensitivity analyses were conducted to evaluate the robustness of the findings. The first analysis examined treatment timing, with RRs estimated separately for patients who received treatment on the index date and for those who received treatment after the index date. The second focused on access to medical specialists, with RRs estimated separately for 3 subgroups: (1) patients diagnosed by a non-specialist and subsequently referred to a specialist, (2) patients whose bladder cancer was diagnosed by a specialist, and (3) patients with a history of any malignancy other than bladder cancer, assuming these patients already had access to a specialist.

## 3 Results

### 3.1 Patient characteristics

**Figure 1:**
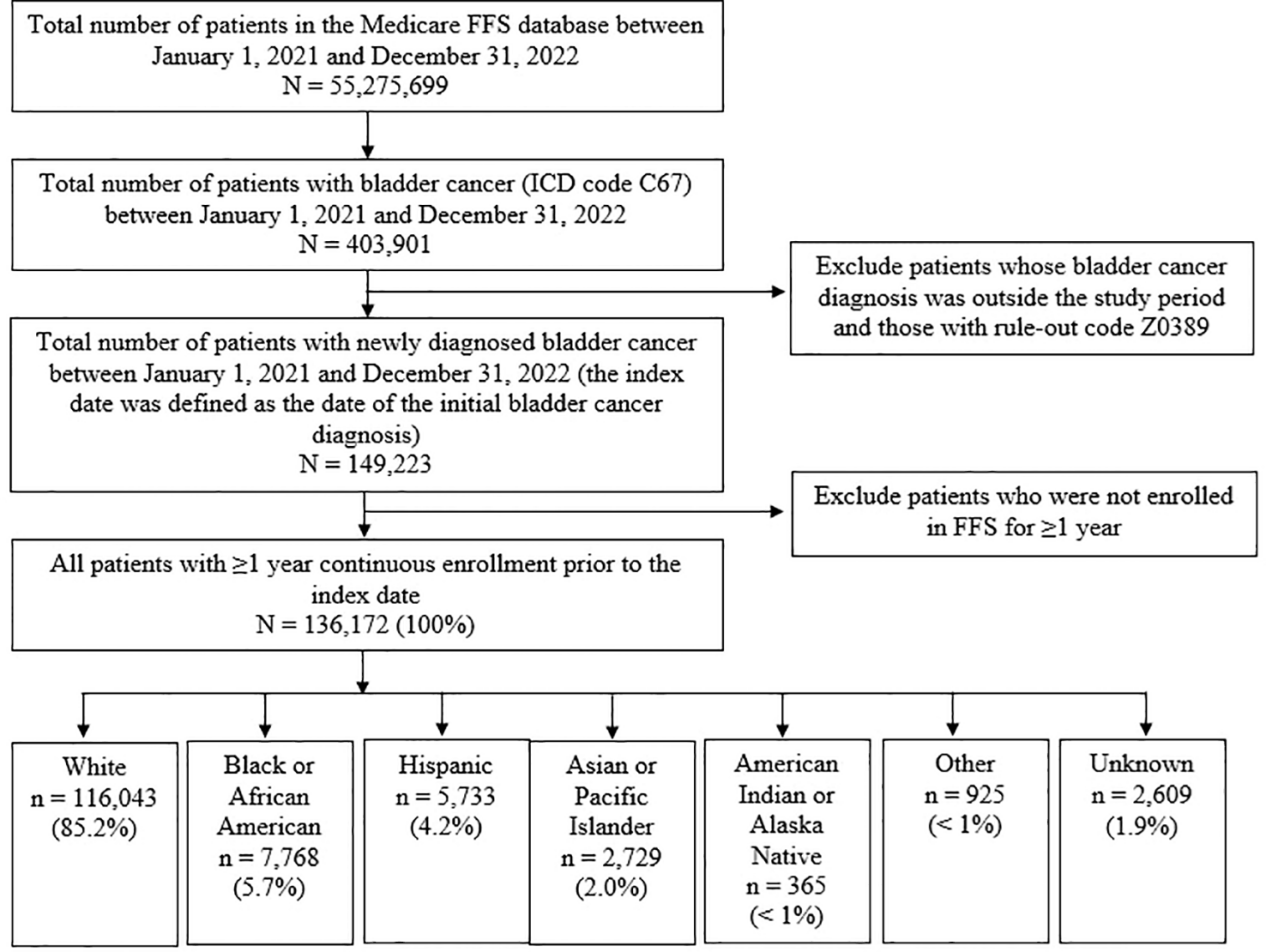
Patient attrition. FFS, fee-for-service; ICD, International Classification of Diseases.

A total of 136, 172 patients met the overall study criteria (**Figure 1**). Among these, 116, 043 (85.2%) were White, 7, 768 (5.7%) were Black or African American, 5, 733 (4.2%) were Hispanic, 2, 729 (2.0%) were Asian or Pacific Islander, 365 (<1%) were American Indian or Alaska Native, and 3, 534 (2.6%) were classified as Other or Unknown race. The median age of all patients was 77 years. Overall, most patients were 65 years or older (96.8%) and male (73.0%), and the majority resided in the South or West region (**Table 1**), with notable geographic variations across racial and ethnic groups. The majority of Black patients lived in the South (55.1%), whereas about one-third of Hispanic patients lived in the South (34.8%) and another one-third in the West (33.1%). Approximately one-half of Asian or Pacific Islander patients (51.3%) and nearly one-half of American Indian or Alaska Native patients (45.2%) lived in the West. Across racial and ethnic groups, most patients resided in urban areas, including 86.0% of White, 92.2% of Black or African Black, 90.0% of Hispanic, 98.5% of Asian or Pacific Islander, and 65.2% of American Indian or Alaska Native patients.

**Table 1:** Patient demographics.

	White N = 116, 043	Black or African American N = 7, 768	Hispanic N = 5, 733	Asian or Pacific Islander N = 2, 729	American Indian or Alaska Native N = 365	Other N = 925	Unknown N = 2, 609
Age category, n (%)							
<65 years	3, 657 (3.2)	675 (8.7)	377 (6.6)	81 (3.0)	41 (11.2)	30 (3.2)	41 (1.6)
65–69 years	17, 085 (14.7)	1, 341 (17.3)	896 (15.6)	375 (13.7)	52 (14.2)	114 (12.3)	596 (22.8)
70–74 years	23, 855 (20.6)	1, 750 (22.5)	1, 125 (19.6)	519 (19.0)	82 (22.5)	168 (18.2)	1, 411 (54.1)
75–79 years	24, 632 (21.2)	1, 429 (18.4)	1, 060 (18.5)	528 (19.3)	77 (21.1)	214 (23.1)	462 (17.7)
80–84 years	20, 885 (18.0)	1, 155 (14.9)	999 (17.4)	483 (17.7)	53 (14.5)	208 (22.5)	45 (1.7)
≥85 years	25, 929 (22.3)	1, 418 (18.3)	1, 276 (22.3)	743 (27.2)	60 (16.4)	191 (20.6)	54 (2.1)
Sex, n (%)							
Female	31, 277 (27.0)	2, 647 (34.1)	1, 776 (31.0)	813 (29.8)	116 (31.8)	221 (23.9)	401 (15.4)
Male	84, 766 (73.0)	5, 121 (65.9)	3, 957 (69.0)	1, 916 (70.2)	249 (68.2)	704 (76.1)	2, 208 (84.6)
Region, n (%)							
Midwest	27, 054 (23.3)	1, 234 (15.9)	400 (7.0)	213 (7.8)	64 (17.5)	129 (13.9)	549 (21.0)
Northeast	25, 647 (22.1)	1, 473 (19.0)	1, 125 (19.6)	578 (21.2)	17 (4.7)	258 (27.9)	726 (27.8)
South	41, 418 (35.7)	4, 284 (55.1)	1, 994 (34.8)	519 (19.0)	119 (32.6)	239 (25.8)	800 (30.7)
West	21, 839 (18.8)	754 (9.7)	1, 899 (33.1)	1, 399 (51.3)	165 (45.2)	293 (31.7)	532 (20.4)
Unknown	85 (<1)	23 (<1)	315 (5.5)	20 (<1)	0	–	–
Urban-rural designation, n (%)							
Urban	99, 812 (86.0)	7, 163 (92.2)	5, 159 (90.0)	2, 687 (98.5)	238 (65.2)	870 (94.1)	2, 372 (90.9)
Rural	16, 146 (13.9)	582 (7.5)	259 (4.5)	22 (<1)	127 (34.8)	49 (5.3)	235 (9.0)
Unknown or missing	85 (<1)	23 (<1)	315 (5.5)	20 (<1)	0	–	–

Per CMS data use requirements, cells with patient counts of 10 or fewer are suppressed to protect beneficiary confidentiality.

### 3.2 Rate ratios for diagnosis

All eligible patients (N = 136, 172) were included in the initial diagnosis analysis. The RRs for initial bladder cancer diagnosis were markedly below 1.0 across all racial and ethnic groups, ranging from 0.403 (95% CI, 0.402–0.403) for Hispanic patients to 0.554 (95% CI, 0.552–0.556) for American Indian or Alaska Native patients (**Table 2**).

**Table 2:** Rate ratios for diagnosis across racial and ethnic groups.

Race	RR	95% CI
White	–	–
Black or African American	0.467	0.467–0.468
Hispanic	0.403	0.402–0.403
Asian or Pacific Islander	0.475	0.474–0.475
American Indian or Alaska Native	0.554	0.552–0.556
Other	0.697	0.695–0.699
Unknown	0.825	0.823–0.826

CI, confidence interval; RR, rate ratio.

### 3.3 Rate ratios for treatment receipt

A total of 59, 966 patients had continuous Part D enrollment between December 2022 and June 2023 (**Supplementary Table 1**). Among them, 44, 117 (73.6%) received any treatment, 41, 676 (69.5%) received non-pharmacological interventions, and 31, 310 (52.2%) received pharmacological treatment. The RRs for receiving any treatment were, again, below 1.0 across all minoritized groups, with the lowest for Hispanic (RR = 0.806; 95% CI, 0.765–0.850) and Black or African American patients (RR = 0.814; 95% CI, 0.775–0.856), followed by American Indian or Alaska Native (RR = 0.832; 95% CI, 0.666–1.039) and Asian or Pacific Islander patients (RR = 0.899; 95% CI, 0.843–0.958; **Table 3**). When stratified by treatment category (pharmacological vs non-pharmacological), similar patterns were observed.

**Table 3:** Rate ratios for treatment receipt across racial and ethnic groups.

Race	RR	95% CI
Any treatment (N = 44, 117)		
White	–	–
Black or African American	0.814	0.775–0.856
Hispanic	0.806	0.765–0.850
Asian/Pacific Islander	0.899	0.843–0.958
American Indian or Alaska Native	0.832	0.666–1.039
Other	0.940	0.840–1.052
Unknown	1.014	0.955–1.076
Pharmacological treatment (N = 31, 310)		
White	–	–
Black or African American	0.765	0.720–0.812
Hispanic	0.720	0.674–0.769
Asian/Pacific Islander	0.893	0.828–0.963
American Indian or Alaska Native	0.732	0.553–0.969
Other	0.921	0.805–1.054
Unknown	1.033	0.963–1.108
Non-pharmacological treatment (N = 41, 676)		
White	–	–
Black or African American	0.786	0.746–0.828
Hispanic	0.782	0740–0.826
Asian/Pacific Islander	0.895	0.838–0.956
American Indian or Alaska Native	0.811	0.644–1.022
Other	0.948	0.844–1.063
Unknown	1.011	0.951–1.075

CI, confidence interval; RR, incidence rate ratio.

For many patients diagnosed with non–muscle invasive bladder cancer, the primary treatment is TURBT. TURBT is considered the standard of care for the initial management and staging of early-stage bladder cancer, and it is typically performed during the initial diagnostic evaluation (22–24). Immediately after TURBT, a single intravesical instillation of chemotherapy (the first line of therapy) is often administered to eliminate any residual tumor cells (22). Our sensitivity analysis by treatment time showed that the RRs for receiving any treatment (e.g., TURBT) on the same day as the initial bladder cancer diagnosis remained well below 1.0 across all racial and ethnic minority groups (**Supplementary Table 2**). For treatment received after the initial diagnosis, the RRs remained below 1.0 for Black or African American (RR = 0.807; 95% CI, 0.746–0.874), American Indian or Alaska Native (RR = 0.889; 95% CI, 0.629–1.258), and Hispanic patients (RR = 0.964; 95% CI, 0.892–1.041), but exceeded 1.0 for Asian or Pacific Islander patients (RR = 1.123; 95% CI, 1.024–1.231).

### 3.4 Rate ratios for specialist visits

During the study period, a total of 116, 950 patients had access to a medical specialist, including 39, 679 patients whose bladder cancer was diagnosed by a medical specialist, 12, 622 patients who were diagnosed by a non-specialist but subsequently referred to a medical specialist, and 64, 649 patients who had a history of a different malignancy prior to their bladder cancer diagnosis. Overall, the RRs for bladder cancer-related specialist visits were lowest for Hispanic patients (RR = 0.891; 95% CI, 0.865–0.918), followed by Black or African American patients (RR = 0.930; 95% CI, 0.906–0.954), and Asian or Pacific Islander patients (RR = 0.978; 95% CI, 0.938–1.019); the RR for American Indian or Alaska Native patients was 1.016 (95% CI, 0.911–1.134; **Table 4**).

**Table 4:** Rate ratios for specialist visits across racial and ethnic groups.

Race	RR	95% CI
All patients who had access to a specialist (N = 116, 950)		
White	–	–
Black or African American	0.930	0.906–0.954
Hispanic	0.891	0.865–0.918
Asian/Pacific Islander	0.978	0.938–1.019
American Indian or Alaska Native	1.016	0.911–1.134
Other	0.999	0.932–1.071
Unknown	1.070	1.028–1.114
Patients who were diagnosed by a specialist (N = 39, 679)		
White	–	–
Black or African American	1.003	0.961–1.047
Hispanic	0.969	0.922–1.019
Asian/Pacific Islander	1.199	1.124–1.279
American Indian or Alaska Native	0.967	0.796–1.174
Other	1.100	0.980–1.233
Unknown	1.241	1.163–1.324
Patients who were diagnosed by a non-specialist and referred to a specialist (N = 12, 622)		
Non-Hispanic White	–	–
Black or African American	1.213	1.130–1.301
Hispanic	1.318	1.219–1.424
Asian/Pacific Islander	1.466	1.319–1.630
American Indian or Alaska Native	1.170	0.851–1.609
Other	1.312	1.086–1.586
Unknown	1.232	1.096–1.386
Patients with a history of a different malignancy before bladder cancer diagnosis (N = 64, 649)		
Non-Hispanic White	–	–
Black or African American	0.834	0.805–0.865
Hispanic	0.767	0.735–0.801
Asian/Pacific Islander	0.758	0.712–0.806
American Indian or Alaska Native	1.017	0.879–1.177
Other	0.883	0.800–0.974
Unknown	0.939	0.886–0.994

CI, confidence interval; RR, incidence rate ratio.

Further analyses stratified by pathways to specialist care (i.e., diagnosed by, referred to, or assumed to already have access to a specialist prior to bladder cancer diagnosis) were performed. Compared with White patients, Black or African American patients had a comparable rate of being diagnosed by a specialist (RR = 1.003; 95% CI, 0.961–1.047), Asian or Pacific Islander patients had a slightly higher rate (RR = 1.199; 95% CI, 1.124–1.279), whereas Hispanic and American Indian or Alaska Native patients had lower rates (RR = 0.969; 95% CI, 0.922–1.019 and RR = 0.967; 95% CI, 0.796–1.174 respectively).

The RRs for being diagnosed by a non-specialist and subsequently referred to a medical specialist were above 1.0 across all minoritized groups, indicating that these patients were more likely to be referred to a medical specialist after being diagnosed by a non-specialist.

Among patients with a history of another malignancy before their bladder cancer diagnosis, the RRs for access to medical specialists were below 1.0 for most racial and ethnic groups (0.834 for Black, 0.767 for Hispanic, 0.758 for Asian or Pacific Islander patients), suggesting they had lower access to oncology specialists.

### 3.5 Time from diagnosis to specialist care and treatment initiation

Compared with White patients, American Indian or Alaska Native and Hispanic patients had longer median times from initial diagnosis to the first specialist visit (28 days for American Indian or Alaska Native patients and 26 days for Hispanic patients vs 23 days for White patients); Black or African American and Asian or Pacific Islander patients had comparable median times (22 days for both groups). However, wide IQRs were observed across all racial and ethnic groups, particularly for Hispanic (8–111 days; **Table 5**) and American Indian or Alaska Native patients (8–121 days), indicating substantial patient-level variability.

**Table 5:** Time from initial diagnosis to the first specialist visit (days) .

Race	Median	IQR
White	23	7–104
Black or African American	22	7–104
Hispanic	26	8–111
Asian/Pacific Islander	22	7–110
American Indian or Alaska Native	28	8–121
Other	30	6–178
Unknown	34	8–148

IQR, interquartile range.

Compared with White patients (25 days), longer median times from initial diagnosis to treatment initiation were observed across racial and ethnic groups, with the longest median time noted among Black or African American patients (31 days) and Hispanic patients (31 days), followed by Asian or Pacific Islander patients (27 days) and American Indian or Alaska Native patients (26 days; **Table 6**). Wide IQRs were evident, again, across racial and ethnic groups, particularly among Black or African American patients (11–94 days), suggesting patient-level variability in when treatment is initiated.

**Table 6:** Time from diagnosis to treatment initiation (days).

Race	Median	IQR
White	25	11–66
Black or African American	31	11–94
Hispanic	31	13–86
Asian/Pacific Islander	27	11–80
American Indian or Alaska Native	26	8–63
Other	24	8–72
Unknown	25	11–65

IQR, interquartile range.

## 4 Discussion

In this large cross-sectional study, we observed notable racial and ethnic differences across the care continuum among Medicare beneficiaries newly diagnosed with bladder cancer. Overall, compared with White patients, minoritized groups—particularly Hispanic and Black or African American patients—had less access to urology and oncology specialists, had lower rates of receiving any treatment, and experienced longer median times from diagnosis to treatment initiation. Hispanic and American Indian or Alaska Native patients also experienced longer median times from initial diagnosis to first specialist visit.

The RRs for initial bladder cancer diagnosis observed in this study were below 1.0 across all racial and ethnic groups, which primarily reflects the lower bladder cancer incidence rates among minoritized populations compared with White individuals. While disease stage at diagnosis could not be assessed in this study because of limitations in the data source, observed delays in diagnosis among minoritized populations are unlikely to contribute meaningfully to the findings in initial diagnosis, as the study captures bladder cancer diagnoses across all stages. Nevertheless, prior studies have shown that compared with White patients, minoritized groups—particularly Black patients—often experience delays in bladder cancer diagnosis and are more often diagnosed at advanced stages (11, 25, 26).

Consistent with prior studies, we also found that minoritized patients in our study, particularly Black or African American and Hispanic patients, had lower rates of receiving any bladder cancer treatment and experienced longer median times from initial diagnosis to treatment initiation than White patients. Differences in RRs were more pronounced for same-day treatment upon diagnosis. For treatment received after initial diagnosis, gaps in RRs narrowed for Hispanic and Asian or Pacific Islander patients but persisted for Black or African American and American Indian or Alaska Native patients. These findings are consistent with results from other studies using different datasets (5, 11, 25, 27, 28). For example, in a large analysis using the National Cancer Database (NCDB; 2004–2016), Hasan et al. found that older and Black patients were significantly less likely to receive treatment for muscle invasive, locally advanced, and metastatic bladder cancer (25). In another NCDB analysis (2004–2017), Buac et al. reported that, compared with White patients, Black and Hispanic patients had significantly increased odds of experiencing treatment delays (5). Similarly, Weinder et al. found that Black patients and those from areas with lower income and educational attainment were less likely to receive treatment and to initiate treatment within 12 weeks of diagnosis (27).

The RRs for access to medical specialists indicate that overall, Black and Hispanic patients were less likely than White patients to have access to oncology specialists for bladder cancer care. Additionally, Hispanic and American Indian or Alaska Native patients were less likely to have their bladder cancer diagnosed by a medical specialist. The longer median times from diagnosis to the first specialist visit for Hispanic and American Indian or Alaska Native patients further indicate that there are barriers to accessing medical specialists for these patients. These findings are consistent with prior research (11, 13). Using SEER-Medicare data (2005-2019), Myers reported that being a Black or Hispanic patient was associated with delays in urologic referrals and visits, a trend that did not improve over time (13). Similarly, a report from the Association of Community Cancer Centers noted that, compared with White patients, Black patients are less likely to receive timely diagnostic evaluation of hematuria, an early sign of bladder cancer for which timely referral for further urologic evaluation is considered a quality benchmark (11).

The differences observed among the racial and ethnic groups in this study likely reflect a complex interplay of healthcare access and delivery, social determinants of health, and individual-level factors (29–33). Although identifying differences in disease stage at diagnosis and survival outcomes were not objectives of this study and were not analyzed, the observed differences in median times from diagnosis to receiving specialty care and to treatment initiation would be expected to affect these outcomes. In this context, prior studies have shown that survival differences between Black and White patients are largely driven by modifiable, system- and care-related factors (8, 9, 26). In a propensity score-weighted hazard analysis, Cole et al. found that although Black patients had significantly higher mortality rates compared with White patients, the differences in overall survival were substantially attenuated after adjusting for treatment- and access-related variables (9). In another study of contrasting healthcare models (a hybrid-payer healthcare model versus an equal-access model), Kotha et al. showed that receiving medical care in an equal-access healthcare system was associated with reduced differences in disease stage at diagnosis and disparities in survival outcomes for African American patients (8). Recent research further suggests that survival gaps between White and Black patients could potentially be reduced when health insurance coverage and neighborhood-level social and economic factors are addressed (4). A study by Johnson et al. evaluated the impact of insurance status and neighborhood-level factors on bladder cancer mortality and found that being uninsured and living in high-poverty areas were associated with worse cancer-specific outcomes among patients with carcinoma-*in-situ* (CIS) and non-muscle-invasive bladder cancer (NMIBC) (4). Notably, the study found that survival gaps in both CIS and NMIBC decreased after accounting for insurance status and neighborhood-level factors (4). Similarly, Silvani et al. reported that greater neighborhood-level disadvantage was associated with higher bladder-cancer specific mortality among patients with NMIBC (34).

This study has notable strengths and provides a contemporary snapshot of differences across the care continuum in real-world practice. First, it leveraged a large Medicare FFS dataset to examine racial and ethnic differences among beneficiaries nationwide. The analysis spans the full continuum of bladder cancer care and provides a comprehensive view of where inequities persist. Moreover, while racial and ethnic disparities in bladder cancer care have been previously described, less was known about disparities in access to specialty care and the time from initial diagnosis to treatment. The findings from this study contribute additional evidence to our understanding of these gaps in care. The sensitivity analyses provide further clarity on how these disparities present in different situations.

As with all real-world observational studies using claims data, the findings from this study should be interpreted with caution. First, the observed initial bladder cancer diagnosis code represents the first administrative claim of a clinical encounter in which bladder cancer was coded and may not reflect the clinician who definitively established the bladder cancer diagnosis. Second, the database lacks information on critical clinical and demographic variables, including cancer stage, disease category, and socioeconomic indicators, which limited our ability to assess potential contributing factors to the differences observed. More importantly, the descriptive nature of this study, coupled with small sample sizes for certain racial and ethnic groups, such as American Indian or Alaska Native patients, may have reduced the precision of estimates. Additionally, some observed differences, such as median time to treatment, are small and may not be clinically meaningful in isolation or substantially change disease trajectory at the individual level. However, when considered along with other disparities in access and treatment, it may reflect broader variations in care pathways. Future studies incorporating inferential methods and clinical outcomes are needed to further evaluate the clinical significance of this finding. Another limitation is that the study population included only Medicare beneficiaries; therefore, the findings may not be generalizable to patients with other types of insurance. Lastly, the cross-sectional design prevents us from establishing causality and does not capture longitudinal outcomes, although these were not objectives of this study.

## 5 Conclusions

In summary, racial and ethnic differences in bladder cancer care were observed in this study. The data provide evidence to support targeted efforts to improve access to care, as well as address challenges along the unique points of the care continuum. Population health decision makers and policy-level stakeholders can use these results to initiate local-level investigations into how healthcare organizations are addressing the unique needs of the bladder cancer care landscape for minoritized groups. Further research is needed to understand the drivers behind these findings and to identify and implement evidence-based strategies to ensure equitable access to care for all patients.

## Data Availability

The data analyzed in this study are subject to the following licenses/restrictions: The data used in this study are Research Identifiable Files from CMS, accessed only by users with a formal Data Use Agreement. Data must be securely accessed through the Chronic Conditions Warehouse Virtual Research Data Center. Requests to access these datasets should be directed to https://www2.ccwdata.org/web/guest/about-vrdc.
