# Supplementary material for: Racial and ethnic differences in bladder cancer diagnosis, treatment, and specialty care access among Medicare fee-for-service beneficiaries: a cross-sectional study
Source: Front Oncol. 2026 Jun 22;16:1797699. doi: 10.3389/fonc.2026.1797699 (PMC13334317; doi:10.3389/fonc.2026.1797699)
Supplement: Supplementary file 1 [file Table1.docx]

**Supplementary Material**

**Table S1:** Number of patients for outcome analyses

|  | **N (%)** |
| --- | --- |
| Total number of patients with ≥12 months of Medicare enrollment between January 1, 2021, and December 31, 2022, who did not have a history of bladder cancer | 47,959,668 |
| All eligible patients | 136,172 (100) |
| Patients eligible for overall treatment analysis (% of all eligible patients) | 59,966 (43.7) |
| Patients who received any treatment (% of patients eligible for treatment analysis) | 44,117 (73.6) |
| Patients who received non-pharmacological interventions (% of patients eligible for treatment analysis) | 41,676 (69.5) |
| Patients who received pharmacological interventions (% of patients eligible for treatment analysis) | 31,310 (52.2) |
| Patients included in the specialist visit analysis (% of all eligible patients) | 116,950 (85.9) |
| Patients included in the time-to-treatment initiation analysis (% of patients eligible for treatment analysis) | 17,139 (28.6) |
| Patients who were referred to a specialist (% of all eligible patients) | 12,622 (9.3) |

**Table S2:** Sensitivity analysis of treatment timing across racial and ethnic groups

| **Race** | **RR** | **95% CI** |
| --- | --- | --- |
| **Patients who received any treatment on the same day as the index date (i.e., the first BC diagnosis date, N=17,139)** | | |
| Non-Hispanic White | - | - |
| Black or African American | 0.819 | 0.769–0.872 |
| Hispanic | 0.708 | 0.660–0.761 |
| Asian/Pacific Islander | 0.759 | 0.695–0.829 |
| American Indian or Alaska Native | 0.797 | 0.597–1.064 |
| Other | 0.873 | 0.752–1.013 |
| Unknown | 1.029 | 0.954–1.110 |
| **Patients who received any treatment after the index date (excluding those who received treatment on the same day as the index date, N=26,978)** | | |
| Non-Hispanic White | - | - |
| Black or African American | 0.807 | 0.746–0.874 |
| Hispanic | 0.964 | 0.892–1.041 |
| Asian/Pacific Islander | 1.123 | 1.024–1.231 |
| American Indian or Alaska Native | 0.889 | 0.629–1.258 |
| Other | 1.048 | 0.883–1.245 |
| Unknown | 0.990 | 0.898–1.091 |

CI, confidence interval; RR, incidence rate ratio.
